# Effects of B2O3 (boron trioxide) on colon cancer cells: our first-step experience and in vitro results

**DOI:** 10.3906/biy-1901-34

**Published:** 2019-06-13

**Authors:** Özgür ALBUZ, Dilek DÜLGER, Beste Çağdaş TUNALI, Feray AYDIN, Selim YALÇIN, Mustafa TÜRK

**Affiliations:** 1 Department of General Surgery, Keçiören Training and Research Hospital, Ankara, Turkey; 2 Department of Medical Microbiology of Basic Medical Sciences, Faculty of Medicine, Karabük University, Karabük, Turkey; 3 Department of Bioengineering, Faculty of Engineering, Kırıkkale University, Kırıkkale, Turkey; 4 Department of General Surgery, 29 Mayıs State Hospital, Ankara, Turkey; 5 Department of Oncology, Faculty of Medicine, Kırıkkale University, Kırıkkale, Turkey

**Keywords:** Boron oxide, L929 fibroblast cells, DLD-1 colorectal adenocarcinoma cells, in vitro

## Abstract

Boron oxide (B2O3) is derived from dehydration of boric acid and is a colorless, semitransparent, crystalline compound that is moderately soluble in water. On the other hand, boron oxide is chemically hygroscopic. This gives the molecule the ability to soak up water and adhere to tissues. Boron oxide can be used locally after tumor debulking in inoperable tumors and especially when the tumor-free margin distance cannot be provided. For all these reasons we aimed to evaluate the in vitro test results of B2O3 in terms of cytotoxicity, genotoxicity, apoptosis, and necrotic effects on L929 fibroblast cells and DLD-1 colorectal adenocarcinoma cells. Our studies demonstrated that boron oxide compounds appear to be highly cytotoxic for both cell lines according to WST cell viability assay (44.22% and 18.36% on DLD-1 and L929, respectively). Although no genotoxic effects were observed, boron oxide compounds showed antiproliferative effects for both cell lines. The prepared boron oxide compounds may hold the potential to be applied locally to the remaining tissue after surgery and further research and evaluation will be needed to determine its effectiveness.

## 1. Introduction

It is evident that cancer is a fatal public health problem worldwide, and colorectal cancers among all cancers are observed with a high frequency in both sexes. According to the 2018 data of the American Cancer Research Institute, colorectal cancer is the third most frequent cancer in males and second most frequent in females. In addition, in 2018, more than 1.8 million new cases were diagnosed globally (AICR, 2018).Prognosis is still limited in patients with metastatic colorectal cancer, despite the emergence of targeted therapy (e.g., cetuximab and bevacizumab) and improvement of other treatment modalities (Nelson and Thorson, 2009). Therefore, it appears that the development of new chemoprophylactic agents is needed for the prevention of colorectal cancer in early stages. Colorectal cancer treatments are still obstructed by delayed diagnosis, metastatic spread through portal blood or lymphatic invasions, iatrogenic recurrence after surgical removal, and failure of specificity in chemo- and radiotherapy (Midgley and Kerr, 1999; Sugarbaker, 1999). Although systemic chemotherapy is possibly the only choice for adjuvant therapy to prevent metastatic spread after resection, local recurrence (e.g., anastomotic leaks) may benefit from local approaches (Sugarbaker, 1999). Compared to other anticancer agents, the use and study of boron as a main agent in colorectal cancers is rare in the literature. Although the methods of administration and the areas of the tumor for which they are applied are different and limited, the research and treatment methods that have boron as a common feature are as follows: boron neutron capture therapy (BNCT) (Wada et al., 2018) uses it for the brain tumor known as glioblastoma multiforme. On the other hand, the hematological anticancer chemotherapeutic agent called bortezomib is authorized for the therapy of cases of multiple myeloma and a hematological anticancer chemotherapeutic agent endorsed for the therapy of cases of mantle cell lymphoma (Chattopadhyay et al., 2015). When we look at the mechanisms of these treatments, the main characteristic of BNCT can be summarized as follows: targeting the destruction of a population of cells identified as cancer cells and minimizing damage to healthy cells. If we look at the physical principles of BNCT, it appears to involve a dual component system [helium-4 (4He) (i.e. α particle) and lithium-7 (7Li) nuclei] on the basis of a nuclear reaction. Low-energy or thermal neutrons are irradiated to the stable boron isotope (10B), and 4He and 7Li are generated. These high-energy charged particles lack the ability to move far. With this treatment, which focuses directly on the tumor cell, the energy is released into the tumor cell, thus directly damaging the DNA and preventing the cells from reproducing (Kahraman, 2004; Yakıncı and Kök, 2016). Since cancer cells in brain tumors are selected and the damage to normal body cells is at a minimal level, this method has become a foremost treatment method (Kahraman, 2004; Yakıncı and Kök, 2016). As mentioned above, bortezomib is used as a chemotherapeutic agent for some hematologic malignancies today; it is a modified dipeptidyl boronic acid analog with antineoplastic activity that acts as a reversible proteasome inhibitor (Chattopadhyay et al., 2015). Another important point that drew our attention while performing this study is that the boronic acid group found in bortezomib is metabolized to the boric acid in the body, and perhaps more importantly, its excretion from the body and the metabolic process were found to be smooth (Pekol et al., 2005). On the other hand, when we look at the structure of boron oxide, it has the formula of diboron trioxide (B2O3), with alternative names such as boric anhydride, boron trioxide, boric oxide, and boric acid anhydride.Boron oxide synthesis can now be done very widely in the chemical industry, and its first production in Turkey was announced in 1994 (Kocakuşak, 1994). In this method, boron oxide is obtained by a three-stage dehydration process with microwave energy. It has been reported that the boron oxides produced are of purity that can be used in many industries, especially chemistry. Boric acid is slowly heated up to 100 °C, followed by a loss of one mole of water, and then converts into metaboric acid (HBO2). It continues to be heated and at 140 °C it loses another mole of water and converts into pyro- or tetraboric acid (H2B4O7). Finally, when the heating is intensified, it loses all its water at red heat (300 °C) and converts into boron trioxide (B2O3), and it is obtained with the same purity (Demir, 2007). Boron oxide forms as colorless, semitransparent, glassy lumps or hard, white, odorless crystals. It has moderate solubility in water at room temperature. Its melting property increases as it is heated (NIH, 2019). It was stated that the structures of boronic acid and boron oxide are very similar and are coordinated with trigonal oxygen, and these two molecules have an indistinguishable spectrum (Gilbert et al., 2000). Bortezomib, a dipeptidyl boronic acid analog, is metabolized to boronic acid. Dipeptidyl boronic acids also have selective and potent proteasome inhibitor properties (Adams et al., 1998; Smoum et al., 2012; Glynn, et al., 2015), as well as a mechanism of inhibition similar to that of NF-κB, suggesting that boron oxide may also have these properties. As a result of a literature review, we found that the different molecular compounds of boron have been studied so far only in limited numbers. Since we saw that boron is not used as an anticancer agent except for some certain types of cancers at the present time, in order to expand the range of use of boron-centered chemotherapeutic agents in medical cancer treatments in the literature, we evaluated the effects of B2O3 (diboron trioxide; boric acid anhydride) molecules as an in vitro chemotherapeutic agent in terms of toxicity, genotoxicity, and apoptotic and necrotic effects on colorectal tumor cells. Our study is original in regards to our experience and results on the effect of B2O3 on colorectal cancer cells, since there is a limited level of knowledge due to the limited application of this compound in solid tumors to date. 

## 2. Materials and methods

### 2.1. Study design 

With this study, we aimed to determine the effect of boron oxide molecules on colorectal cells. The contents of 4 separate samples containing equal amounts of boron oxide (B2O3) were as follows: the first group contained boron oxide (B2O3), which is easily supplied in the chemical industry; the second group included boron oxide produced in laboratory conditions and boric acid, which can be easily obtained from the chemical industry; the third group included boron oxide that we produced in laboratory conditions; and the fourth group contained boron oxide and boric acid, both obtained from the chemical industry. Each of these 4 separate samples were divided into subgroups containing 20 µg/mL, 10 µg/mL, 5 µg/mL, and 2.5 µg/mL of the molecule, respectively. Group I: Sample contained B2O3 obtained from the chemical industry.Group II: Sample contained B2O3 produced in the laboratory + boric acid obtained from the chemical industry.Group III: Sample contained B2O3 produced in the laboratory. Group IV: Sample contained boric acid and B2O3 obtained from the chemical industry. (The amount of boric acid in Group II and Group IV is equal in both samples.)B2O3 synthesis was done by dehydrating boric acid in 3 steps in a dry oven under laboratory conditions (at 100 °C, 140 °C, and 300 °C, respectively). As is clearly stated in the literature, metaboric acid (HBO2) is formed after the first stage, when the temperature reaches 140 °C; then one mole of water is lost and the molecule is transformed into pyro- or tetraboric acid (H2B4O7), and finally, in the final heating phase (300 °C), by completely losing water, boron trioxide (B2O3) is formed (Demir, 2007; Taşgın, 2018). For calculation of dehydration, molar loss is calculated by following the weight change of the water with losses as a result of each heating process.Boron oxide was produced in the laboratory, commercially obtained, or freshly prepared as dilutions with deionized water and applied in the first 24 h; thus, no stock or stabilization problems were experienced. However, B2O3 is hygroscopic at room temperature and moderately soluble in water (Ediz and Özdağ, 2001). As with bortezomib, propylene glycol was chosen as a solvent system for boron oxide due to its ease of liquid stabilization. 

### 2.2. Cell cultures for fibroblast and colon cancer cells

Normal L929 (derivative of strain L mice fibroblast cells) and DLD-1 (ATCC CCL-221) human colorectal adenocarcinoma cells and also CHO (Chinese hamster ovary) cells as a standard cell line for genotoxicity were placed in flasks containing DMEM (Dulbecco’s modified Eagle’s medium for L929 and CHO cell lines) and RPMI 1640 (Roswell Park Memorial Institute 1640 medium developed from monolayer human leukemic cells in suspension for DLD-1 cell line) with L-glutamine, 10% fetal bovine serum FBS), and 1% antibiotics and remained in a CO2 incubator conditioned with 5% CO2 at 37 °C for 48 h. For procuring cells, the cell culture medium was removed and the cells were treated with trypsin-EDTA (0.05%), and then cells were transferred to 15-mL Falcon tubes and centrifuged at 2500 rpm for 2 min.Removing the supernatant, the cells were counted and required amounts of cells were used in the prospective studies.

### 2.3. Cell viability with WST assay

L929 and DLD-1 cells were placed in 96-well plates (5 × 103 cells/well) containing DMEM and RPMI 1640, respectively, with L-glutamine, 10% FBS, and 1% antibiotics. After the plates were kept in a CO2 incubator (37 °C in 5% CO2) for 24 h until the cells were attached to the bottom of the plate, the culture medium was replaced with fresh medium and then the cells were treated with different concentrations (20-2.5 µg/mL) of samples (Groups I-IV), and the cytotoxicity on L929 and DLD-1 cells was evaluated by WST1 colorimetric assay. After finishing the experiment, the plates were instantly scanned in an ELISA Microplate Reader (BioTek, USA) at 440-nm wavelength and the percentage of cell viability for each group was assessed with the definition of the control cell viability as 100%.The variables are expressed as mean ± standard error as calculated from 3 separate experiments. Significant differences in the mean values were evaluated by t-test, and values of P < 0.01 and P < 0.05 were considered statistically significant.

### 2.4. Analysis of apoptotic and necrotic cells (live/dead double staining)

Double staining (DS) of Hoechst dye with propidium iodide (PI) is one of the methods that provide the characterization and assessment of apoptosis and necrosis in culture with the principle of scoring cell nuclei.L929 and DLD-1 cells were cultured in 96-well plates (5 × 103 cells/well) in DMEM and RPMI 1640 separately, in complementary material with 10% FBS and 1% antibiotics at 37 °C and 5% CO2 moist atmosphere conditions overnight. Cells were then treated with different concentrations (20-2.5 µg/mL) of samples (Groups I-IV) for 24 h. When the incubation was completed, the cell culture media were separated from all wells and 70 µL of the DS solution, containing 10 mL of PBS, 500 µL of Hoechst 33342 (200 µg/mL), 100 µL of PI (100 µg/mL), and 100 µL of RNAse A (10 mg/mL), was added to the cells. The plate was then coated with aluminum foil and incubated for 15 min in an incubator. Both apoptotic and necrotic cells were counted with an inverted fluorescence microscope (Leica DM IL, Germany), via DAPI [apoptotic cells were stained with Hoechst (green)] and FITC [necrotic cells were stained with PI (red)] filters, respectively. The final data were presented as percentages of green and red cells after counting 100 cells from each sample over three randomly selected areas.Variables are stated as mean ± standard error as calculated from 3 separate experiments.The differences between the mean values of the measurement results were calculated by t-test and P-values are based on P < 0.05. 

### 2.5. Micronucleus (MN) assay for genotoxicity

An in vitro MN assay was performed to determine the frequency of MN in the cytoplasm of interphase cells. The presence of MN in the cell is an indicator of chromosomal damage. In this part of the study, to verify the potential genotoxic effects of samples, CHO cells (Chinese hamster ovary is a standardized cell line for genotoxicity assays) were seeded in 12-well plates (104 cells/well) in 1 mL of DMEM medium supplemented with 10% FBS and 1% antibiotics. The plates were then preserved in a CO2 incubator for 24 h. When the cells were inoculated to the base of the plate, the cell culture medium was substituted with fresh medium and a 10 µg/mL concentration of all samples (in the final density) was placed into the wells. A final density of 200 µg/mL ethyl methane sulfonate (EMS) was used as a positive control. Following 44 h of culture (the time required for 1.5-fold cell cycle time according to the OECD Test Guideline 487), cytokinesis blockage was applied by including 3 µg/mL of cytochalasin-B.When the 72-h incubation period was complete, ice-cold KCl (0.075 M) treatment for 2 min at room temperature was followed by adding a few drops of 4% formaldehyde to preserve the cytoplasm and fixation with methanol-glacial acetic acid solution (Meth-Ac, 3:1 mixture). Wells were incubated in each solution for per 3 min. Fixation was followed by painting with PI for MN. After painting for 5 min, binucleated cells were observed for each sample with a fluorescence microscope for MN, nucleoplasmic bridges, and nuclear buds.

### 2.6. Cell proliferation assay using the xCELLigence system

The xCELLigence system is a microelectronic biosensor system for cell-based assays providing dynamic, real-time, label-free evaluation of proliferation, viability, and cytotoxicity of cells (Urcan et al., 2010). This technology was used to determine the proliferation of L929 and DLD-1 cells according to the guidance of the instructions of the provider (Roche Applied Science, Switzerland). The xCELLigence RTCA analyzer measures resistance variations in cell culture media found in E-plate wells (with gold microelectrodes integrated into the bottom of the wells) in the incubator and determines cell viability and motility against toxic potency. The more cells that accumulate on the electrodes, the bigger the increase in electrode resistance. However, cell number is not a factor determining the impedance. Other factors like cell interaction strength with the electrodes and cell morphology affect the impedance measurement. Therefore, electrode resistance, which is exhibited and recorded as cell index (CI) values, reflect the degree of the biological status of monitored cells, including the cell number, cell viability, and morphologic and adhesiveness ability (Ramis et al., 2013). In this work, for background reading, 100 µL of DMEM or RPMI 1640 was added to a 96-well E-plate and the plate was placed into the xCELLigence incubation system for 10 min for stabilization of the temperature of the plate and the device. Followed by a background reading for 1 min, the E-plate was taken out of the system and L929 and DLD-1 cells (5 × 103 cells/well) were inoculated in the plate. The E-plate was placed one more time for incubation and the system ran for 10 min to confirm whether the wells were read or not. For cell joining and growth, the plate was read for 24 h. When 24 h was completed and the cells had reached the growth phase, the E-plate was removed from the incubator and the medium was discarded. Then a ﬁnal volume of 200 µL of medium containing various concentrations of samples from Groups I-IV (final concentrations of samples were 10 µg/mL and 5 µg/mL) was added to cells. Afterwards, the plate was left for incubation for approximately 72 h to observe the cell index and growth status of the cells.

### 2.7. Hematoxylin and eosin (H&E) staining

L929 and DLD-1 cells (104 cells/well) were incubated in 48-well plates containing DMEM and RPMI 1640. After incubation with 20 µg/mL and 5 µg/mL concentrations of the samples (Groups I-IV) for 24 h, the cells dyed with H&E were examined with a light microscope for cellular and nuclear morphologies (Leica, DMI 6000, Germany). Cell membrane integrity was impaired when the specimens were applied to the cells at 20 µg/mL concentration, so the images obtained in the case of application of 5 µg/mL concentrations of the specimens are shared in Section 3.

## 3. Results

### 3.1. WST assay for cytotoxicity 

**Figure 1:**
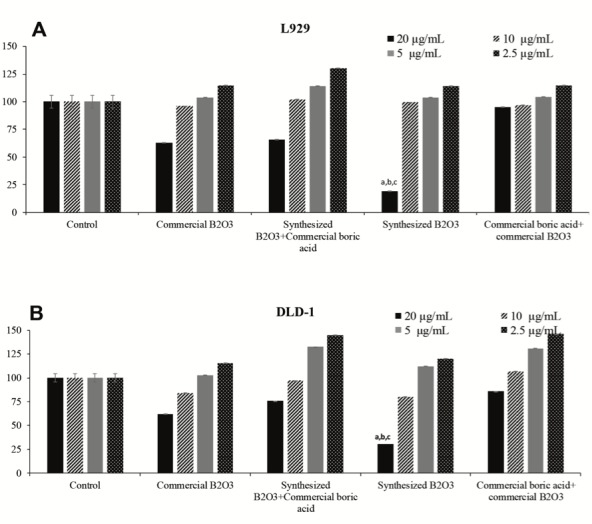
The cytotoxic effects of differing concentrations of specimens on L929 (A) and DLD-1 cells (B) as clarified by WST assay.

With this research, the cytotoxicities of the all groups’ samples (Groups I-IV) were tested against L929 and DLD-1 cells. The 20, 10, 5, and 2.5 µg/mL concentrations of all group samples were incubated with separate cell lines for 24 h in cell culture medium as described above. Additionally, wells containing only cells specified as controls were also tested in cell culture medium. Subsequently, the wells were read at 440 nm with an ELISA plate reader. The percentage of viable cells was estimated at various concentrations (20, 10, 5, and 2.5 µg/mL) against % cell viability (shown in Figures 1A and 1B). In terms of WST results, the concentrations of specimens did not have a notable effect on cell mortality, except the 20 µg/mL concentration of samples in both cell lines. An increase of the samples’ concentrations caused a slight decrease of cell viability in comparison to control cells. When both types of cells (DLD-1 and L929) were treated with the highest concentration of Group III samples (20 µg/mL), cell viability was found to be significantly lower (P < 0.01) compared to the other groups. Similar results were obtained with DLD-1 cells. Briefly, the lowest cell viability at the highest concentration was detected with the Group III sample (45.22%), which was statistically significant (P < 0.05) compared to the other groups. On the other hand, the highest cell viability was seen at the lowest concentration for the sample from Group I (115.75%) on DLD-1 cells. The cell viability (%) established based on mean absorbance values at 440 nm is presented. Data are given as mean ± standard error as calculated from separate experiments. Lowercase letters mark statistically significant differences.

### 3.2. Analysis of apoptotic and necrotic cells (live/dead double staining)

**Figure 2:**
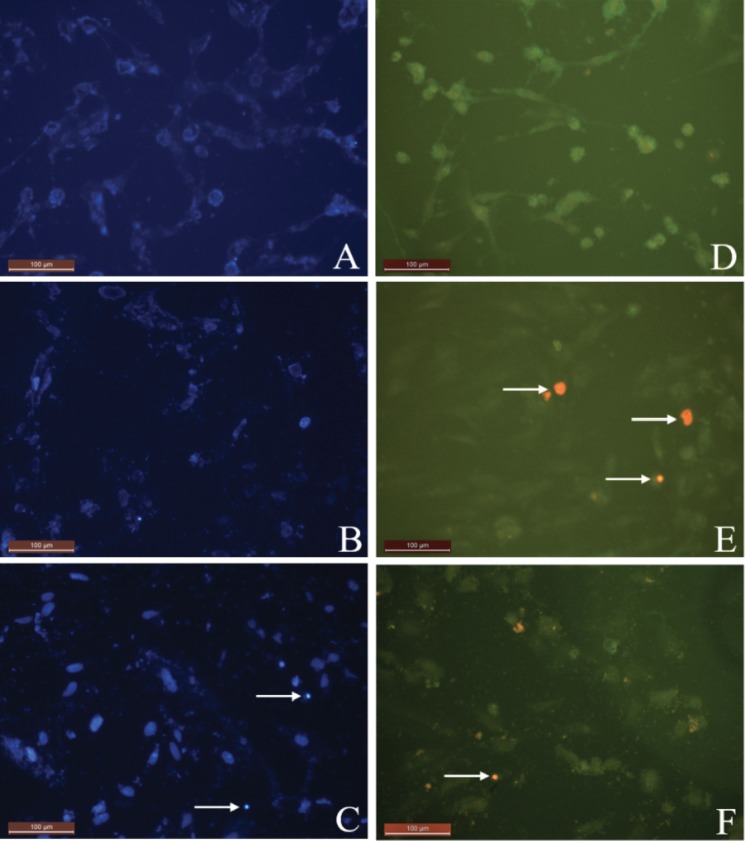
Apoptotic and necrotic L929 fibroblast cell images procured from the double-staining procedure. A) Control group (Hoechst 33342); B) Group I sample (Hoechst 33342); C) Group III sample (Hoechst 33342); D) control group (PI); E) Group II sample (PI); F) Group IV sample (PI). Cells were treated with 10 μg/mL concentrations of samples. Determination of the scale was done according to a distance of 100 μm (200× magnification).

**Figure 3:**
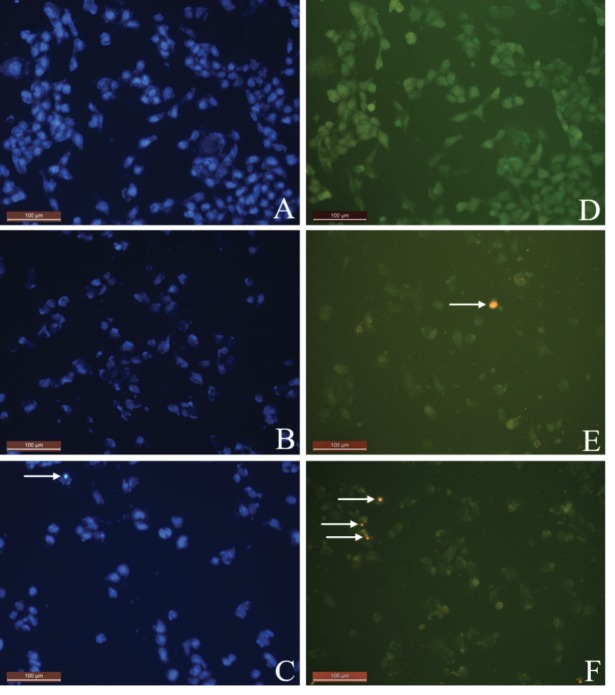
The double-staining procedure was used for obtaining apoptotic and necrotic DLD-1 colon cancer cell images. A) Control group (Hoechst 33342); B) Group II sample (Hoechst 33342); C) Group IV sample (Hoechst 33342); D) control group (PI); E) Group I sample (PI); F) Group III sample (PI). Cells were treated with 10 μg/mL concentrations of the samples. The scale displays a distance of 100 μm (200× magnification).

**Figure 4:**
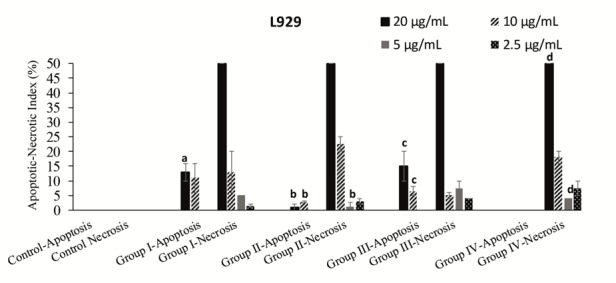
Apoptotic and necrotic indexes acquired for L-929 fibroblast cell cultures after incubation with different concentrations of group samples. Data are specified as mean ± standard error as calculated from 3 different experiments.

**Figure 5:**
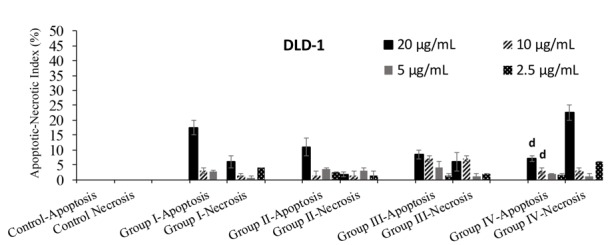
The necrotic indexes obtained for L929 fibroblast cell and DLD-1 colon cancer cell cultures respectively after incubation with various concentrations of extracts. Data are mean ± standard error as evaluated from 3 separate experiments.

To research the apoptotic and necrotic effects and the relationship with the morphological changes for all samples on L929 and DLD-1 cells, Hoechst 33342 fluorescent stain was used in this study.Respectively, all samples’ dilutions were arranged at concentrations between 20 and 2.5 µg/mL with fresh medium and applied to the cultivated normal and cancer cell cultures. The interaction was followed continuously for each group’s samples with cells for 24 h. In the double-staining method, both apoptotic and necrotic cells images are given (Figures 2A-2F and 3A-3F). The Hoechst 33342 fluorescent stain is used to fix DNA and obtain a dark blue color for cell nuclei when observed by the DAPI filter with a fluorescent microscope. The control group included only medium. There was no apoptosis as there was no morphological difference in the cell nuclei of the control group (Figures 2A and 3A).No significant apoptotic effect was detected in the Group I samples (Figure  2B and 3B).Apoptotic cell nuclei broke apart and had shapeless borders and brighter images compared to the nonapoptotic cells (apoptotic cells are shown with arrows in Figures 2C and 3C).When we looked at the control group, we did not see any morphological variations in the cell nuclei (Figures 2A and 2D). In some of the treated samples (samples of Group I, Figure  2B, and samples of Group III, Figure  2C), the apoptotic cell nuclei were stained with a stronger blue (white arrows) (fluorescence compared to nonapoptotic cells) (Figure  2C). A necrotic cell appearance was obtained from the double-stain method via PI fluorescent stain. PI, a fluorescent molecule, was chosen in the double-staining procedure to define necrotic cancer cells. Although this dye passes through the dead cell membranes and causes cell nuclei staining in red, it does not pass through the living cells’ membranes under fluorescent light with the FITC filter. The L929 and DLD-1 cells were dyed with PI fluorescent dye in double-staining solution. Necrotic cell images are given in Figures 2D, 2F, 3D, and 3F. Normal cell nuclei were found as green when investigated by FITC fluorescent filter, but the images of the control group showed no morphological differences in cell nuclei (Figures 2D and 3D). The nuclei of necrotic cells were determined to be red in color in wells treated with all samples, especially at high concentrations (20 and 10 µg/mL), by the utilized fluorescent microscope (Figures 2E, 2F, 3E, and 3F).In the control group, no morphological differences were seen in the cell nuclei (Figures 3A-3D). In some treated samples (Group II sample, Figure  3B, and Group IV sample, Figure  3C), at the final concentration (10 µg/mL), the apoptotic cell nuclei were stained with a stronger blue (white arrows) (fluorescence compared to nonapoptotic cells) (Figure  3C). Necrotic cell images were obtained via the double-staining method using PI fluorescent stain. An apoptotic effect was clearly observed for the 20 µg/mL concentration of Group I and Group III samples for L929 cells and all group samples for DLD-1 treated with the same concentrations, given as % apoptotic indexes in Figures 4 and 5. The apoptosis of the cells treated with the Group I sample (20 µg/mL) was significantly less (P < 0.05, indicated as “a”) than the necrosis for the same group and concentration. In all other groups, apoptotic effects of different concentrations were also found to be significantly different than necrotic effects [P < 0.05; 20, 10, and 5 µg/mL concentrations of Group II (indicated as “b”), 20 and 10 µg/mL concentrations of Group III (indicated as “c”), 20 and 5 µg/mL concentrations of Group IV (indicated as “d”)]. As a consequence, the increase in concentration caused a general enhancement of apoptosis.The lowest necrotic effect was observed at the concentration of 2.5 µg/mL for Group I and Group III samples, and high necrotic effects were observed especially at the concentrations of 20 µg/mL and 10 µg/mL of samples for L929 fibroblast cells and for 20 µg/mL concentrations of Group I and Group IV samples for DLD-1 colon cancer cells. Figures 4 and 5 show the apoptotic and necrotic cell indexes of L929 and DLD-1 cells acquired from the double staining of Hoechst and PI.The findings showed that at the highest concentration (20 µg/mL) of the group samples, except for the Group IV sample, none of the samples caused a significant apoptotic index, but necrotic indexes were >50 for all samples at the highest concentration (20 µg/mL) for the L929 cell line.

### 3.3. Micronucleus assay for genotoxicity

**Figure 6:**
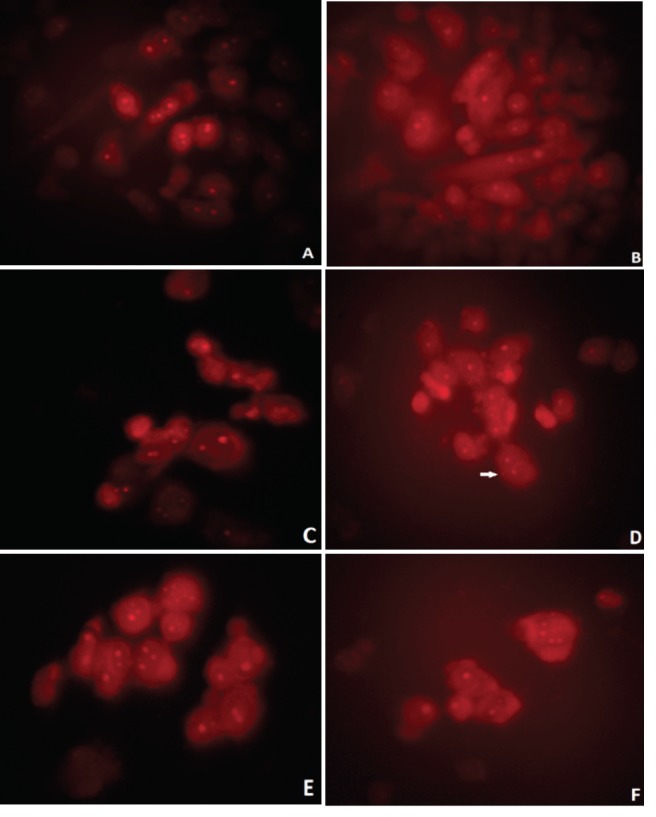
Micronucleated binucleated cell visualization status from micronucleus assay via PI fluorescent stain. Fluorescence microscopy image of (A) CHO cells not processed with samples (nonpositive control group). Cells were not micronucleated. (B) Cells reacted with EMS at a concentration of 200 μg/mL (positive control); (C) cells exposed to the Group I sample at a concentration of 10 μg/mL; (D) Group II sample at a concentration of 10 μg/mL, where white arrow shows micronucleus; (E) Group III sample at a concentration of 10 μg/mL; (E) Group IV sample at a concentration of 10 μg/mL. Photographs were obtained by FITC filter using a Leica inverted fluorescent microscope (400× magnification).

In relation to the MN assay in CHO cells, no significant increase in the frequency of micronucleated binucleated cells was observed compared to the controls (Figure  6). Additionally, no further increase in micronucleus induction was exhibited beyond the concentration of 10 µg/mL for all specimens.

### 3.4. Cell proliferation assay using xCELLigence system

**Figure 7:**
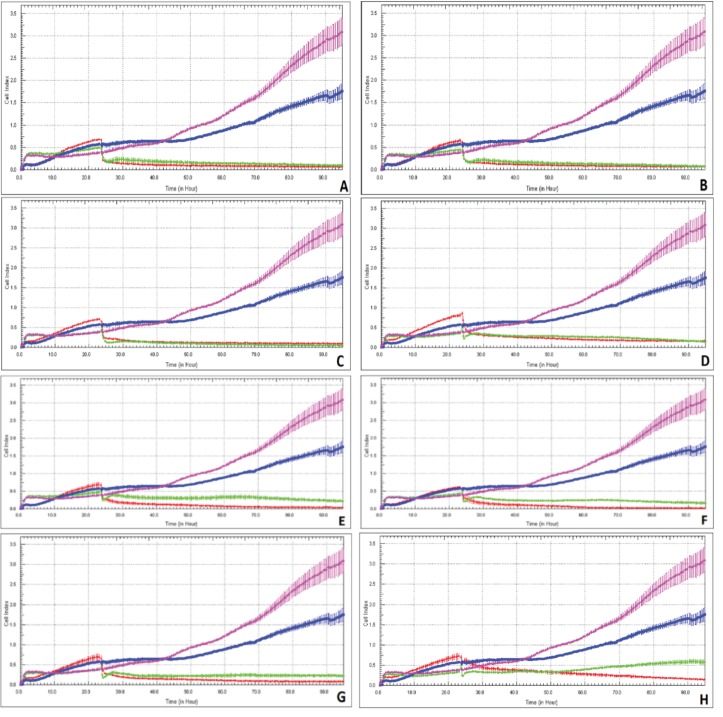
Dynamic monitoring of L929 and DLD-1 cells treated with 5 and 10 μg/mL concentrations of samples using the real-time analyzer (RTCA) SP. A) Group I sample (10 μg/mL); B) Group II sample (10 μg/mL); C) Group III sample (10 μg/mL); D) Group IV sample (10 μg/mL); E) Group I sample (5 μg/mL); F) Group II sample (5 μg/mL); G) Group III sample (5 μg/mL); H) Group IV sample (5 μg/mL). Red line: L929 cells treated with sample; blue line: L929 cells not treated with sample (control group); green line: DLD-1 cells treated with sample; pink line: DLD-1 cells not treated with sample (control group).

Cell inhibition and antiproliferative effects of individual samples of Groups I-IV on L929 and DLD-1 cells were analyzed by WST1 and RTCA assays, respectively. Figures 1 and 7 exhibit the result of the tests obtained from cytotoxicity and cell proliferation assays. It can be seen in Figures 7A-7F that in cells treated with samples of Groups I, II, III, and IV, proliferation was reduced (>50%) in both cell cultures (L929 and DLD-1) for both concentrations of 5 and 10 µg/mL, and there was no observed proliferative effect.

### 3.5. Hematoxylin and eosin staining

**Figure 8:**
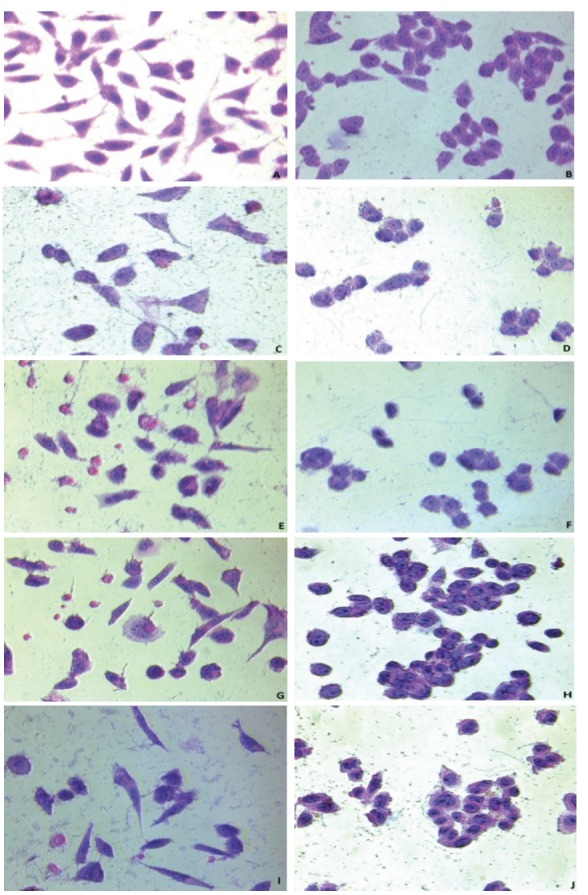
Light microscopy micrographs of H& E-stained L929 and DLD-1 cells. (A) L929 cells show morphological shapes such as relatively cylindrical (control group); (B) DLD-1 cells are more round than L929 cells (control group); (C) L929 cells treated with Group I sample (5 μg/mL); (D) DLD-1 cells treated with Group I sample (5 μg/ mL); (E) L929 cells treated with Group II sample (5 μg/mL); (F) DLD-1 cells treated with Group II sample (5 μg/mL); (G) L929 cells treated with Group III sample (5 μg/ mL); (H) DLD-1 cells treated with Group III sample (5 μg/mL); (I) L929 cells treated with Group IV sample (5 μg/mL); (J) DLD-1 cells treated with Group IV sample (5 μg/ mL). Images were taken under a Leica inverted light microscope (400× magnification).

In the last century, H&E stains have been commonly used and they are still important for defining different tissue types and morphologic changes. This histologic dye has been successfully used for many years as it works efficiently with various fixatives and plays a key role with its extensive range of cytoplasmic, nuclear, and extracellular matrix features. With a poorly understood reaction, hematoxylin has a dark blue-purple color and smudges nucleic acids. On the other hand, eosin is pink and it dyes the proteins nonspecifically. Interestingly, in specific tissue, nuclei are stained blue, but the cytoplasm and extracellular matrix have varying degrees of pink smudges (Fischer et al., 2008). After staining with hematoxylin and eosin of L929 and DLD-1 cells, which were treated with 20 and 5 µg/mL concentrations of samples, morphological variability could be observed between the cells (5 µg/mL concentrations of samples), as in Figure  8. This may be related to the cytotoxic effects of the samples on the cells.

## 4. Discussion

Overall, there are three main methods for the treatment of cancer. These methods are the administration of chemotherapy, the administration of radiotherapy, and surgery for resectable tumors. Surgery is naturally more effective in early-stage tumors. However, cancer cells can always remain as residue at the cellular level (Nedunchezhian et al., 2016). At this stage, the efficacy and side effects of chemotherapy and radiotherapy gain importance. To date, some studies have examined the anticancer effects of boron in medicine and its effect on general human health according to daily exposure. Unfortunately, boron was not used in the medical field until 2003 with the use of bortezomib (Velcade), an FDA-approved proteasome inhibitor, for various hematological malignancies (Richardson, et al., 2003). Following this, PT-100 (Val-boroPro, Talabostat), which focus on dipeptidyl peptidases such as fibroblast activation protein, has been started to be used in the field of medicine in the framework of efforts to develop other boron-centered compounds. In 2004, a phase II clinical trial of PT-100 was initiated in the treatment of advanced non-small-cell lung cancer (Cunningham, 2007). The compound, which was put on hold in 2007 due to efficacy problems, has recently been considered as a therapeutic drug for many metastatic cancers such as kidney cancer, pancreatic adenocarcinoma, non-small-cell lung cancer, and chronic lymphocytic leukemia, and phase II clinical studies are being carried out (Jansen et al., 2013). Okondo et al. reported that Val-boroPro (PT-100, Talabostat) induced strong antitumor immune responses in similar cancer models (Okondo et al., 2018). In the same study, they reported that Val-boroPro (PT-100, Talabostat) acts as an immunostimulator in caspase-1-dependent cell death, called pyroptosis (Okondo et al., 2018). Pyroptosis is one of the forms of nonapoptotic cell death, but it is different from necrosis (Tait et al., 2014). Namely, pyroptosis acts on caspase 1-dependent cell death. Thus, a war is started between host and pathogen mechanisms (cancer, inflammatory diseases, microbial infections, etc.) for pyroptosis and this is important in terms of cell viability. Pathogen mechanisms aim to inhibit the pyroptosis (Bergsbaken et al., 2009). Although the reasons and the results are not fully understood, it has been pointed out that IL-1b and IL-18 maturation may be associated with caspase-1 and thus pyroptosis occurs for the release of mature, inflammatory cytokines. It is also emphasized that as a final response to the triggering of invasive factors that defeat the host defense mechanism, it controls the spread by killing the host cells. Sendler et al. also stated that pyroptosis is a highly inflammatory type of cell death and involves the activation of nuclear factor-kB. In addition, they emphasized that it can be an alternative programmed cell death pathway in cases where apoptosis does not work (Sendler et al., 2016). Val-boroPro (PT-100, Talabostat) activates this pathway. This subject, which requires further research, has also attracted our attention and raises the question of whether or not the necrotic cellular structure that we have determined in our study is a mechanism of pyroptosis for boron oxide. Considering the mechanisms of action of bortezomib, which we mentioned earlier, first we can say that it reversibly inhibits the 26S proteasome, a large protease complex (Geniş and Coşar, 2018). The inhibition of this proteasome function has also come up as a strong strategy for anticancer therapy. Bortezomib has been reported to induce apoptosis in cancer cells as an inhibitor of proteasome, and even green tea effectively antagonizes its ability to induce apoptosis in cancer cells by the bonds with the boronic acid portion of the bortezomib, with its epigallocatechin-3-gallate structure (Glynn et al., 2015).In fact, the tendency of bortezomib and boron components to form covalent bonds allows the boron atom to function as a strong Lewis acid (electron pair acceptor), due to the presence of an empty p-orbital. As a result of this, thanks to the empty p-orbital, which is open to a pair of electrons, it can easily form covalent bonds with the hydroxyl groups of biological nucleophiles such as serine in the enzyme structures, carbohydrates, and nucleic acids (Baker et al., 2009), and it was reported that this makes it possible to create new compound structures that can reduce necrosis. In contrast to the antagonistic effect of green tea for bortezomib, *Curcuma longa* has agonistic effects with different borate-containing compounds. These agonistic effects arise from some inhibitors enzymes such as serine protease, aspartic protease, metalloprotease, p-glutamyl transpeptidase, and threonine-based and cysteine protease inhibitors (Smoum et al., 2012). On the other hand, a transcription factor, NF-κB, can be induced by controlling the signal activation steps. This factor improves carcinogenesis and cancer progression by controlling many genes involved in inhibition of apoptosis in immuno-inflammatory responses. Colorectal cancer metastases were found to be inhibited by inhibition of NF-κB (Feng et al., 2016). Bortezomib is also capable of specifically inhibiting nuclear factor NF-κB at this stage. Bortezomib delays tumor growth in vivo and increases the cytotoxic effects of radiation and chemotherapy, as also shown by Engür and Dikmen 2017. Sun et al. 2012 also showed that sodium butyrate suppresses colon carcinogenesis by triggering colon cancer cell apoptosis, dependent on the store-operated Ca2+ entry (SOCE). One of the interesting points here is that although Sun et al. claimed that the mechanism of this reaction is like a puzzle, they indicated that 2-APB (2-aminoethoxydiphenyl borate) contributes to the mechanism of action of sodium butyrate with pharmacological blockade of SOCE (Sun et al., 2012). The definitive role of diphenylboronic anhydride (2APB) in the SOCE blockade was first demonstrated in the literature by Dobrydneva and Blackmore 2001. As we mentioned in Section 1, boron oxide is a dehydrated state of boric acid, bortezomib is metabolized to boric acid in the body at the same time, and the effect of boron oxide with its hygroscopic properties is thought to be higher on cells when applied. All these factors led us to research boron oxide. In addition, when we look at the daily boron levels for human beings, the tolerated upper level limit defined by the US Institute of Medicine’s Food and Nutrition Board is 20 mg/day for adults (Trumbo et al., 2001).The World Health Organization set the initial recommended level of intake as 13 mg/day, but then they increased this value and identified it as 0.4 mg/kg, or 28 mg/day of boron for a human being at 70 kg (WHO, 1998). The European Union has determined the maximum tolerable level of intake as 10 mg/day based on body weight. The acceptable daily intake for boron was determined as 0.16 mg of boron/kg per day by the 2013 EFSA Panel (EFSA, 2013). Therefore, boron safety limits are not within a narrow spectrum like those of other chemotherapeutics.The cytotoxic effect of the samples prepared at different concentrations was investigated by WST test on both L929 and DLD-1 cancer cell lines. We determined that the cytotoxic activity was 55.78% for DLD-1 and 81.64% for L929 when the highest concentration of the Group III sample was used, indicating that cancer cells are more resistant than normal cells. Furthermore, we think that a cytotoxic effect of 55.78% for DLD-1 is important.Boron nitride nanotubes (BNNTs) are highly water-soluble nanostructured boron nitride compounds that can be used in BNCT. Singh et al. 2016 examined the cytotoxic effects of nanostructured boron nitride in HeLa (cervical cancer) and human breast adenocarcinoma (MCF-7) cells in their study, and after administration of a 2 mg/mL dose of the drug in 24 h they found 60% and 45% cytotoxicity, respectively. In normal cell culture (HEK-293), they found this rate at the same dose and time as 30% (Singh et al., 2016). The results of our cytotoxicity tests are similar.When cell death was investigated by dual staining test, which is done through apoptotic or necrotic pathways, compared with the control groups, the apoptotic cell index was found to be higher for L929 (35%) than for DLD-1 (8.5%). Similar results were also obtained for necrotic cell indices. The lower apoptosis in colon cancer cells (DLD-1) than in human fibroblast cells (L929) is also due to the fact that cancer cells with induced cytotoxic effects are more aggressive and resistant.Li et al. 2017 reported that empty boron nitride (BN) nanospheres increased apoptosis in prostate cancer as a result of controlled crystallization, and reduced cell viability. When the results of this study by Li et al. were evaluated, it was found that DU145 prostate cancer cells had increased early and late apoptosis fractions of 16.96%-32.68% and 14.42%-52.41%, respectively, after exposure to BN spheres with different crystallinity properties (25 µg/mL) for three days (Li et al., 2017). Another similar study was conducted by Rocca et al. 2016, and they confirmed the potential toxicity of pectin-coated boron nitride nanotubes (P-BNNTs) in terms of proliferation of RAW 264.7 mouse macrophages, inductance of oxidative stress, and apoptosis/necrosis phenomena in the range of 0-50 µg/mL (Rocca et al., 2016). After 24 h of incubation with P-BNNTs, the measurements of necrotic, early apoptotic, and late apoptotic cells ranged between 4.4% and 11.5%, 0.3% and 2.2%, and 1.5% and 2.5%, respectively (Rocca et al., 2016). In our study, in which we evaluated the effect of B2O3 on colorectal cancer cells, which we believe will contribute to the literature, we have not observed any changes in the morphological cells that we worked on. Additionally, due to the detection of only one micronucleus formation in the second sample, we see encouraging results for our future studies. As a result, we think that stopping cell proliferation in both the DLD-1 and L929 cell lines is a favorable outcome that inhibits cancer development. Boron oxide maybe applicable as a systemic, but it needs to be researched with in vivo experimental next steps. Although it has an undesirable necrotic effects on control cell line L929, it can be utilized locally in intraoperative cancer surgery because of its induced apoptosis and tumor tissue destruction. In the present study, we identiﬁed our boron oxide-based original molecule, which can be effective on colon cancer, in terms of stimulating apoptosis and necrosis without any genotoxic effects.Boric acid anhydride (boron oxide) may play an important role in terms of intraoperative application for local use after tumor debulking for inoperable tumors or especially when the tumor-free margin distance cannot be provided. If this is confirmed by in vivo tests, it will be an important step towards a novel drug. As a next step, we are planning to build an animal model for colorectal cancer with boron trioxide (B2O3). We believe that this boron-based compound should be focused on with its anticancer effects, and multicenter national organization is crucial.
